# Influence of learning styles on the practical performance after the four-step basic life support training approach – An observational cohort study

**DOI:** 10.1371/journal.pone.0178210

**Published:** 2017-05-22

**Authors:** Hanna Schröder, Alexandra Henke, Lina Stieger, Stefan Beckers, Henning Biermann, Rolf Rossaint, Saša Sopka

**Affiliations:** 1 Department of Anaesthesiology, University Hospital RWTH Aachen University, Aachen, North Rhine-Westphalia, Germany; 2 Aachen Interdisciplinary Training Centre for Medical Education, Medical Faculty RWTH Aachen University, Aachen, North Rhine-Westphalia, Germany; 3 Department of Internal Medicine, Hermann-Josef-Hospital, Erkelenz, North Rhine-Westphalia, Germany; 4 Department of Internal Medicine, Agaplesion Elisabethenstift, Darmstadt, Hesse, Germany; Waseda University, JAPAN

## Abstract

**Background:**

Learning and training basic life support (BLS)—especially external chest compressions (ECC) within the BLS-algorithm—are essential resuscitation training for laypersons as well as for health care professionals. The objective of this study was to evaluate the influence of learning styles on the performance of BLS and to identify whether all types of learners are sufficiently addressed by Peyton’s four-step approach for BLS training.

**Methods:**

A study group of first-year medical students (n = 334) without previous medical knowledge was categorized according to learning styles using the German Lernstilinventar questionnaire based on Kolb’s Learning Styles Inventory. Students’ BLS performances were assessed before and after a four-step BLS training approach lasting 4 hours. Standardized BLS training was provided by an educational staff consisting of European Resuscitation Council-certified advanced life support providers and instructors. Pre- and post-intervention BLS performance was evaluated using a single-rescuer-scenario and standardized questionnaires (6-point-Likert-scales: 1 = completely agree, 6 = completely disagree). The recorded points of measurement were the time to start, depth, and frequency of ECC.

**Results:**

The study population was categorized according to learning styles: diverging (5%, n = 16), assimilating (36%, n = 121), converging (41%, n = 138), and accommodating (18%, n = 59). Independent of learning styles, both male and female participants showed significant improvement in cardiopulmonary resuscitation (CPR) performance. Based on the Kolb learning styles, no significant differences between the four groups were observed in compression depth, frequency, time to start CPR, or the checklist-based assessment within the baseline assessment. A significant sex effect on the difference between pre- and post-interventional assessment points was observed for mean compression depth and mean compression frequency.

**Conclusions:**

The findings of this work show that the four-step-approach for BLS training addresses all types of learners independent of their learning styles and does not lead to significant differences in the performance of CPR.

## Introduction

In cardiopulmonary resuscitation (CPR), high-quality external chest compressions (ECC) represent the core element to generate blood flow in order to maintain heart and brain oxygenation and have the greatest influence on patient outcome after in- or out-of-hospital cardiac arrest [1,2]. Therefore, recurring training in resuscitation is unavoidable for both medical professionals and laypersons. The European Resuscitation Council (ERC) recommends frequent assessment and refresher courses including the application of feedback devices and self-directed learning strategies [1]. In order to adapt to these recommendations, strategies are needed to avoid poor quality ECC in the long term. [3,4] Therefore, it is essential to investigate learning habits and educational methods to improve practical skill performance in basic life support (BLS). Peyton’s four-step approach as an instructional strategy to teach technical skills to learners remains the only method recommended by the ERC for resuscitation training even in ERC guidelines before 2010 [1]. Originally constructed by Peyton for a 1:1 student-teacher ratio [5], it was adapted for resuscitation training over ten years ago [6] and is used in international courses for trauma or advanced life support course concepts. It is based on four steps of instruction:

1. *Demonstration*: skill demonstration at normal speed without explanation.
2. *Deconstruction*: repetition of the skill’s steps with elaborate explanation and encouragement of the learner to ask questions.
3. *Comprehension*: learner’s explanation of the steps of the skill and demonstrator’s instruction on the correct performance. Necessary corrections from the demonstrator and repetitions of this step are performed until full understanding is achieved.
4. *Performance*: the learner practices the skill under observation and receives feedback.

The four-step approach has been successfully modified in various settings with video and media assistance [7].

**Fig 1:**
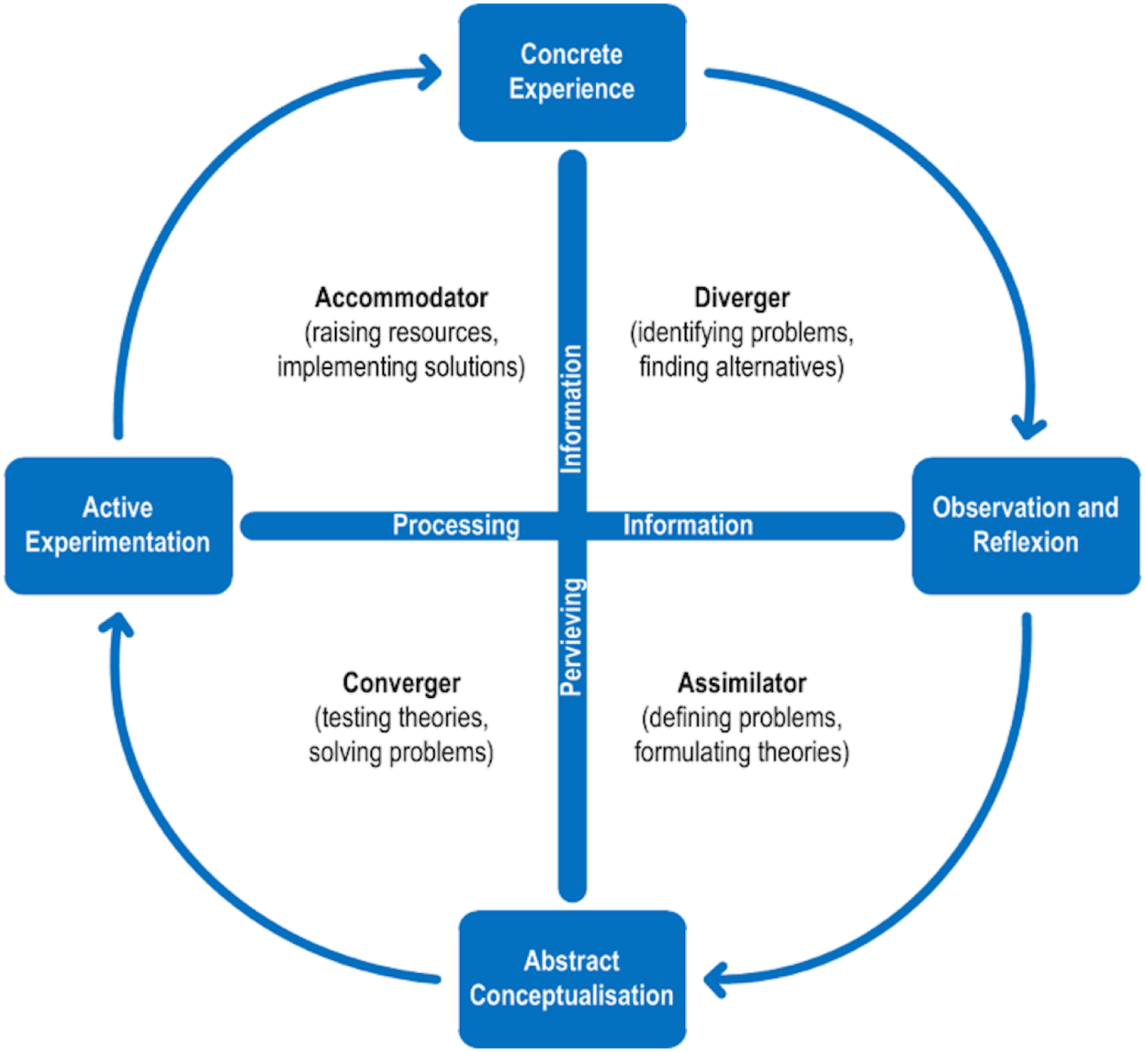
Kolb's model of learning styles. Adapted from Kolb, D. A.: Experiential Learning: Experience as the Source of Learning and Development, Englewood Cliffs, NJ: Prentice Hall, 1984:42).

The relevance of learning models and consideration of learning styles in medical education have been critically discussed and evidence is still lacking regarding whether adapted teaching methods and instructions provide a significant benefit [8]. Different versions of Kolb’s Learning Style Inventory have been used to categorize learners’ approaches to study and performance [9;10;11]. According to Kolb, learning is based on perceiving information and transforming it through reflection or active experimentation and practice [12]. The dimensions of how information is gained and how it is processed define the four learning styles defined by Kolb and underline his theory of experiential learning:

*diverging (DIV)*: processing a concrete experience that leads to reflective observation (experiencing and watching), *assimilating (ASM)*: the development of abstract theories from observed findings and reflections (watching and thinking), *converging (CON)*: transformation of an abstract theory or concept into active experimentation and action (doing and thinking), and *accommodating (ACC)*: active experimentation that leads to concrete experiences (doing AND feeling) (Fig 1).

Until now, there has been no evidence on whether the four-step approach adequately addresses different learning styles in the case of BLS training. The question of whether our standard training concept for BLS, based on the four-step-approach, needs to be adapted to learning styles of the target group was the objective of the present study.

### Objectives

The major aim of this study was to assess the impact of learning styles (adapted from those defined by Kolb) on BLS performance after training using a standardized four-step BLS training approach within a population of laypersons and to identify whether the four-step approach is (dis)advantageous for any type of learner.

## Methodology

### Study design

In this prospective observational study, randomized groups of participants were first asked to complete a German-adapted questionnaire based on Kolb’s Learning Styles Inventory and then tested in a mock cardiac arrest scenario as single rescuers (pre-interventional assessment). All groups then received a standardized 4-hour BLS training course from educational staff consisting of several ERC-certified ALS providers under the supervision of an advanced life support (ALS) instructor. The training consisted of a 15-min slide-based tutorial. For the practical training, the instructors equally applied the four-step approach. The four-step approach consisted of a real-time BLS demonstration by the instructor, followed by a slow demo with explanation and time for questions (deconstruction), instructor performance under guidance from the learning group (comprehension), and demonstration by the learners and free time to practice [7,8]. Equal time was permitted for training and practice as well as support and feedback from the instructors during the training. The post-interventional assessment took place one week after the training, using exactly the same scenario as before. Because of the observational nature of this study, no sample size determination was carried out. It was not possible to assume how many of the included persons would be assigned to any learning style. For that reason, three generations of first-year students were included.

### Participants

Between 2007 and 2010, first-year medical students (n = 371) from the Medical School of RWTH Aachen University, Germany, were invited to participate in the study during their first two weeks of introductory lessons at the medical school. The participants were defined as nonprofessionals as they had no previous medical education or concrete training in resuscitation skills. Students with previous medical emergency training such as paramedics, medical technicians, or nurses were considered healthcare providers and excluded from the study. The final study group consisted of 334 students who completed the questionnaire and participated in the four-step-approach training intervention as well as the pre- and post-assessments. The participants were randomized into groups of 10–12 individuals and included learners of all learning styles.

The local ethics committee of the medical faculty of RWTH Aachen University approved the study (number EK 100/12). The participants were informed about the assessment of their CPR performance as part of the scientific purposes of the study and written informed consent was obtained from each student before their first evaluation. Neither evaluators nor the students themselves had information about results of their questionnaire and their assigned learning styles.

### The German “Lernstilinventar” questionnaire

The participants were categorized into different learner types based on their responses to the German “Lernstilinventar” (LSI) questionnaire by Haller and Nowack from Göttingen [13]. The LSI is a 5-page questionnaire in German based on the English Learner Style Inventory by Kolb. The participants completed the questionnaire manually and independently. Afterward, the scores were calculated and the participants were further categorized into the four types following the manual for analysis equal to the original version.

### Measurements and data acquisition

All pre- and post-intervention scenarios were performed equally in instructions and evaluation following a standardized testing protocol. The participants had no chance to see others’ performances and did not receive any feedback on their performance during the assessment.

The scenarios were always constructed to be single-rescuer CPR scenarios requiring initial assessment, breathing control, and immediate ECC in combination with mouth-to-mouth ventilation. The scenarios were terminated after 180 s. The standardized test setup consisted of a manikin dressed in a zippered jacket (Skillreporter Resusci^®^ Anne, Laerdal, Stavanger, Norway) and placed on the floor in a supine position. Laerdal PC-Skillreporting Software (Version 1.3.0, Laerdal, Stavanger, Norway) was used for data acquisition of the ECCs. A certified ERC ALS instructor supervised the data recording.

### Performance data

The practical endpoints were in line with former guidelines [2]. Therefore, the average compression depth within the range of 40–50 mm and rate of ECC within 90–110 min^-1^ were chosen as the parameters for quality CPR performance. The “time to start CPR” (the duration until the first compression) was recorded for each scenario by the instructor. Finally, a standardized checklist was used to observe and assess the participants’ general approach to the scenario and manikin. The checklist measured self-security, assessment of consciousness, breath control, emergency call, and delay until starting ECC.

### Statistical data analysis

For the main endpoints, two-way repeated measures analysis of variance (repeated measures ANOVA) was calculated to investigate the effect of learning style (four levels: DIV, ASM, CON, or ACC), time (2 levels: baseline or after one week) and the resulting two-factor interaction of learning style and time. Suitable contrasts were formulated and tested to compare the effects at a point of time within learning styles or between time points for a single learning style. The continuous variables are summarized as means and corresponding standard deviations (±). The categorical data are presented as percentages. All tests were two-sided and were assessed at the 5% significance level. We did not adjust the significance level to account for multiple tests because of the exploratory nature of the parallel study hypotheses. The analyses were performed using SAS^®^, V 9.2 (SAS Institute, Cary, NC, USA).

## Results

### Study population

Overall, a total of 371 students completed the entire study. It was not possible to assign learning styles to 37 subjects because of incomplete survey responses. From the 334 valid subjects, 30% (n = 101) were male and 70% (n = 233) were female. The four learning styles were distributed as following: diverging, 4.8% (n = 16); assimilating, 36.3% (n = 121); converging, 41.3% (n = 138); and accommodating, 17.7% (n = 59) (Table 1).

**Table 1:** Demographic data of the study group.

Participants (n = 334)		
**Age (years)**	mean:	21.2
	SD	±3.8
	range	17–42
**Sex**	female:	233 (70%)
	male:	101 (30%)
**Learning style**	diverging	16 (4.8%)
	converging	138 (41.3%)
	assimilating	121 (36.3%)
	accommodating	59 (17.7%)

### Observed endpoints

#### Performance data

Evaluation of the overall performance records at baseline assessment revealed a mean compression depth of 44.1 ± 11 mm, mean compression frequency of 99.4 ± 24.3 min^-1^, mean time to start CPR of 31.65 ± 15.4 sec, and mean initial assessment score of 3.1 ± 1.7 points. Within the entire group of participants, the post-interventional assessment showed significantly improved performance for compression frequency (105.4 ± 16.4 min^-1^) and assessment by checklist (6.8 ± 1.6 points). A positive tendency was observed in the time to start CPR, but the difference was not significant (29.1 ± 9.9 sec). Post-interventional compression depth was comparable to the baseline (43.4 ± 8.6 mm) (Table 2).

**Table 2:** Overview of the performance results.

Performance measurement			Overall (n = 334)			
			mean	SD	p1 (overall)	p2 (sex)
Compression depth (mm)	PRE	Overall	44,1	11	0.129	0.002*
		Male	48,7	10,1		
		Female	42,1	10,8		
	POST	Overall	43,4	8,6		
		Male	44,1	9,7		
		Female	42,9	8,5		
Compression frequency (min^-1^)	PRE	Overall	99,4	24,3	0.019*	0.002*
		Male	108,2	24,7		
		Female	95,5	23,1		
	POST	Overall	105,4	16,4		
		Male	107,8	18,9		
		Female	103,9	16,5		
Time to start CPR (sec)	PRE	Overall	31,6	15,4	0.458	0.837
		Male	31,9	15,6		
		Female	31,4	15,3		
	POST	Overall	29,1	9,9		
		Male	31,6	9,8		
		Female	28,1	9,7		
Assessment by checklist (points)	PRE	Overall	3,1	1,7	0.001*	0.091
		Male	3,4	2		
		Female	3	1,6		
	POST	Overall	6,8	1,6		
		Male	7,1	1,5		
		Female	6,7	1,7		

Compression depth in mm, compression frequency in min^-1^, time to start CPR in seconds, assessment by checklist in points.

* indicates a significant difference, p1: overall pre/post difference; p2: difference of performance regarding sex.

**Fig 2:**
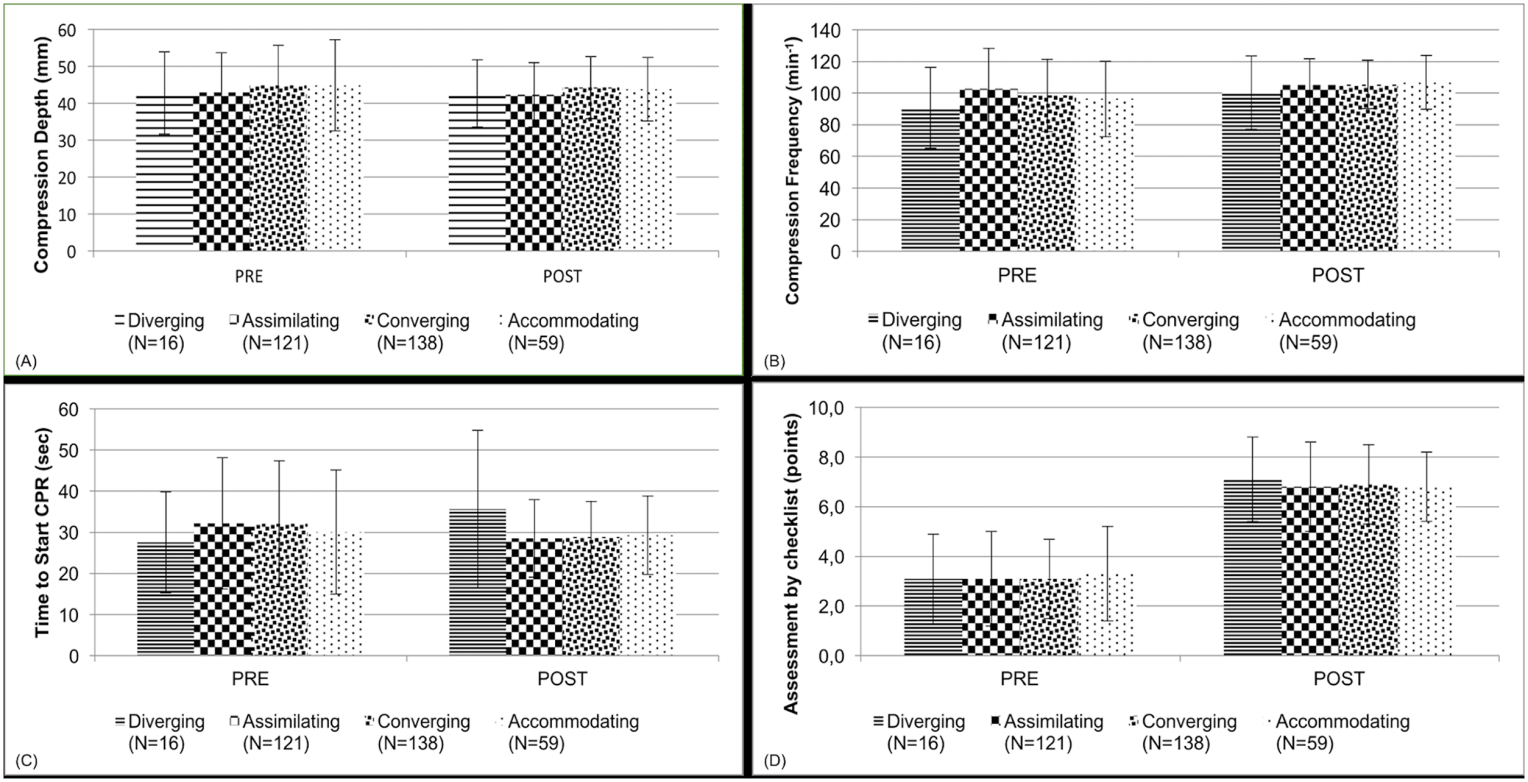
**Performance of learning style groups:** (A) compression depth (mm), (B) compression frequency (min^-1^), (C) Time to start CPR (s), (D) Assessment by checklist (points).

Evaluation of the differences between the four groups of learning styles at baseline revealed no significant differences in compression depth (p = 0.87), compression frequency (p = 0.31), time to start CPR (p = 0.54), or checklist-based assessment (p = 0.71). Furthermore, comparison of the degree of improvement according to learning styles between pre- and post-interventional assessments did not show significant differences for any of the measurement endpoints (Fig 2A–2D).

#### Influence of sex

At baseline, performance differences were observed in compression depth between male (mean: 48.7 ± 10.1 mm) and female (mean: 42.1 ± 10.8 mm) participants (p < 0.01). Similarly, there were significant sex differences in compression frequency, (108.2 ± 24.7 vs. 95.5 ± 23.1 min^-1^, respectively). There were no significant differences at baseline according to sex in the time to start CPR and the points of the initial checklist-based assessment (Table 2).

While the post-interventional assessment of male participants revealed mean compression depth and frequency decreases to 44.1 ± 9.7 mm and 107.8 ± 18.9 min^-1^, respectively, the female participants improved in compression depth (42.9 ± 8.5 mm) and frequency (103.98 ± 16.5 min^-1^). Therefore, we observed sex differences in the development of CPR performance for compression depth and frequency (p = 0.002).

## Discussion

The main results of our study show that learning styles did not influence learning CPR performance. The standardized four-step BLS training approach addressed all learning styles, with no observed differences between the learners' performance. The major aims of the present study were to assess the impact of learning styles on the BLS performance after a standardized four-step approach within a population of laypersons and to identify if this approach sufficiently addressed all learner types.

To our knowledge, this is the first study to compare performance according to learning styles in the context of BLS training. As the four-step-approach concept in BLS training has been shown to foster skill acquisition and retention of CPR performance [6;14], we investigated the impact of this teaching method based on Kolb’s learner style theory. Although different BLS training approaches have been evaluated [15;16], no studies have reported the potential need for BLS training based on individual learning styles.

Overall, the mean baseline data revealed the ECC to be in the lower range of the former ERC guidelines (depth 40–50 mm, frequency 90–110 min^-1^); however, but within the ranges in the current guidelines (depth: 50–60 mm, frequency 100–120 min^-1^). Besides the improved frequency, the magnitude of the standard deviation decreased after training and performance was adjusted. In particular, the participants' approach to a CPR scenario, including victim assessment, placing an emergency call, and starting ECC, were improved following the four-step BLS training approach. These aspects are extremely important in BLS training in addition to high-quality ECC to ensure the best possible patient outcome.

A wide range of surveys have been developed to assess learning style. The common critique of Kolb’s inventory is that it mainly addresses psychometric conditions. Based on these critiques, the tool has been revised in the decades since it was first proposed in order to improve its reliability and validity and address measurement issues [17;18]. Kolb’s LSI has been used in previous studies on medical education to analyze target groups [9] or to investigate the efficacy of style-based educational designs [10;11;18].

Previous studies have reported the distributions of learning styles in groups of up to 177 medical students and residents. The assimilating and converging styles were predominant within medical students, while the converging and ACC learner styles were predominant within surgical residents [9]. Our findings in 334 participants also indicated the assimilating and converging styles to be predominant within medical students, as would be expected in the field of applied natural sciences. We confirmed that the DIV learner style appears to be underrepresented within the medical population.

In our opinion, Peyton’s four-step approach naturally employs different strategies to address learner habits. The first three phases (demonstration, deconstruction, and comprehension) address different cognitive pathways. The demonstration requires the learner to be attentively watching, while the deconstruction requires simultaneous active listening and reflection. The learner *assimilates* what is shown and what is explained. The comprehension phase, when the learner guides the demonstrator to perform the skill, connects and, for the first time, applies what the learner has understood. One could argue that the learner must merge what he has learned so far; this phase is repeated until everyone in the target group has fully understood the skill. The final phase of practical training represents the active experimentation that leads to concrete experience. It *accommodates* what has been learned and assures the achievement of the defined learning outcomes. As Kolb’s theory of learning styles also has been described as a cycle and process of learning, [6] Peyton’s approach aligns and overlays with Kolb’s concepts. Previous findings have demonstrated the benefit of adapting learning methods and instructions to learner styles on an individual basis, focusing on students’ areas of concern [18]. In contrast to this, various groups have pointed out that intensive review of data does not provide sufficient evidence to support style-based learning instructions [8;10]. We believe that the four-step approach already addresses different learner styles with varying habits and preferences. Our observations of the lack of differences in skill performance verified that individual learners with certain learning styles are not disadvantaged by the four-step approach. We, therefore, conclude that, for practical skills training, the elements of demonstration, explanation, and deconstruction followed by practice and experience adequately address the audience no matter the learning style of the group.

In the wide field of practical skills training, the four-step approach has become a fundamental instrument for instructive teaching over the last decade. The current research questions include whether simple skills such as laryngeal mask (LMA) insertion and percutaneous needle puncture cricothyroidotomy can be delivered with minimal time and personnel effort through a modified two-step approach [11;19]. Advisors to the ERC argue that a simple skill such as LMA insertion “makes learning relatively independent from the teaching technique” if a skill is easy to perform, very easily taught, and easily learned [20]. However, due to the poor availability of comparable data, no superiority over the four-step approach has been shown, especially regarding BLS training. Future comparative research should assess style-based learning instructions to determine whether they are superior to four-step approach training.

### Limitations

This study has several limitations. First, as the study investigated the influence of a training method on the performance improvement of different learning styles, the results are still transferable to recent ERC guidelines; even with the adoption of the 2015 updated ERC guidelines, the suggestions for BLS training methods did not undergo major changes during the revision. The observed study group was not representative of sex (30.2% male and 69.8% female participants), but for laypersons at the beginning of medical studies and groups have been comparable. As we have reported previously [21;22], most first-year medical students have no specific preparation after graduation from secondary school and are therefore considered laypersons in the field of resuscitation. A power calculation was not conducted for the current study, but previous observational CPR studies reported an enrollment of 27–40 participants per group with a power of 0.8 to detect a 5% difference [23;24]. We also must consider the possible effects of repeated testing. Finally, in addition to learning preferences and teaching method, additional influencing factors such as learning environment or assessment have been shown to affect learning. [25–27]. These factors were not investigated in our study; however, we did create a standardized learning environment to assure the comparability of our data. There was no skill retention testing after six months.

## Conclusion

The findings of this work demonstrate that the four-step approach for BLS training addresses all types of learners independent of their learning styles and does not lead to significant differences in the performance of CPR. Especially for training practical skills such as CPR, the elements of demonstration, deconstruction, and comprehension, followed by practice and experience, are crucial for achieving homogenous learning effects. Prospective studies should analyze whether other instructional learning style-adapted methods are superior to four-step-approach training.

## Supporting information

S1 Table Dataset—Influence of learning styles on BLS training. Learning Styles 1 = diverging, 2 = assimilating 3 = converging, 4 = accommodating; sex 1 = male, 2 = female; mDT_mm = mean depth of compressions; mFreq = mean frequency of compression; IA_Zeit = time to start CPR; IASSges = checklist points for Initial Assessment, 1 indicates before intervention, 2 indicates after intervention.
(XLSX)

S1 Checklist STROBE checklist. (PDF)
